# TGFβ and CCN2/CTGF mediate actin related gene expression by differential E2F1/CREB activation

**DOI:** 10.1186/1471-2164-14-525

**Published:** 2013-08-01

**Authors:** Noel Faherty, Helen O’Donovan, David Kavanagh, Stephen Madden, Gareth J McKay, Alexander P Maxwell, Finian Martin, Catherine Godson, John Crean

**Affiliations:** 1 UCD School of Biomolecular and Biomedical Sciences, UCD Conway Institute, University College Dublin, Belfield, Dublin, Ireland; 2 UCD School of Medicine and Medical Sciences, UCD Conway Institute, University College Dublin, Belfield, Dublin, Ireland; 3 National Institute for Cellular Biotechnology, Dublin City University, Glasnevin, Dublin, Ireland; 4 Nephrology Research Group, Centre for Public Health, Queen’s University Belfast, Belfast, UK

**Keywords:** TGF beta, CTGF/CCN2, Actin, CREB, E2F1

## Abstract

**Background:**

CCN2/CTGF is an established effector of TGFβ driven responses in diabetic nephropathy. We have identified an interaction between CCN2 and TGFβ leading to altered phenotypic differentiation and inhibited cellular migration. Here we determine the gene expression profile associated with this phenotype and define a transcriptional basis for differential actin related gene expression and cytoskeletal function.

**Results:**

From a panel of genes regulated by TGFβ and CCN2, we used co-inertia analysis to identify and then experimentally verify a subset of transcription factors, E2F1 and CREB, that regulate an expression fingerprint implicated in altered actin dynamics and cell hypertrophy. Importantly, actin related genes containing E2F1 and CREB binding sites, stratified by expression profile within the dataset. Further analysis of actin and cytoskeletal related genes from patients with diabetic nephropathy suggests recapitulation of this programme during the development of renal disease. The Rho family member Cdc42 was also found uniquely to be activated in cells treated with TGFβ and CCN2; Cdc42 interacting genes were differentially regulated in diabetic nephropathy.

**Conclusions:**

TGFβ and CCN2 attenuate CREB and augment E2F1 transcriptional activation with the likely effect of altering actin cytoskeletal and cell growth/hypertrophic gene activity with implications for cell dysfunction in diabetic kidney disease. The cytoskeletal regulator Cdc42 may play a role in this signalling response.

## Background

The actin cytoskeleton is highly dynamic, constantly being remodelled in living cells and may become dysfunctional in various kidney diseases, including diabetic nephropathy (DN) [1-4]. *In vitro*[5,6] and *in vivo*[7] studies have shown disruption of the cytoskeleton in renal mesangial cells exposed to high extracellular glucose and growth factors. Similarly, the migration of epithelial cells, such as those undergoing epithelial to mesenchymal transition, is dependent on the reorganisation of the cytoskeleton. *In vitro*, actin mediated contractile changes are often modelled using cell migration responses; migration being characterised by the same polarisation, protrusion, adhesion and extensive dynamic cytoskeletal and microtubular reorganisation seen in disease related cytoskeletal dysfunction [8]; and regulated by complex signalling networks initiated and integrated by integrins and other receptors [9-11].

In the context of DN, it is accepted that both Transforming Growth Factor-β (TGFβ) and one of its downstream effectors Connective Tissue Growth Factor (CCN2/CTGF) play roles in regulating the pathogenesis of this fibrotic kidney disease. CCN2 has been reported to regulate TGFβ superfamily signalling in various contexts including renal mesangial/epithelial cell dysfunction [12,13] and DN [14]; coordinate expression of TGFβ and CCN2 has been demonstrated in glomerulonephritis and DN [15] and the cooperative nature of TGFβ and CCN2 in the promotion of fibrosis in animal models has been established [16].

The role of CCN2 and TGFβ in the modulation of mesangial cell migratory response was first described by Blom and colleagues [17] and subsequently characterised as being dependent on ERK MAP kinase, Protein Kinase B and Protein Kinase C [5,18]. Intriguingly, the interplay between these factors and the migratory capacity of the cells was altered in the presence of high glucose levels, attributed to changes in cell polarisation caused by dysregulation of the PKC-ζ/GSK3β signalling axis [19]. We previously reported that the combination of TGFβ and CCN2 induce a gene expression profile characterised by the differential expression of a unique subset of genes in human mesangial cells [13]. In the present study we interrogated this subset of genes and identified a role for the transcription factors CREB and E2F1 in mediating altered actin and cytoskeletal related dynamics. This transcriptional programme is evidenced by a non-vectorial migration profile *in vitro* when mesangial cells are treated with both growth factors, reflecting an altered cell phenotype that may be considered profibrotic and hypertrophic. The data from this study provide useful information for the characterisation of cytoskeletal dysfunction in fibrotic disorders associated with increased expression of TGFβ and CCN2 [13].

## Results and discussion

### Co-treatment of mesangial cells with TGFβ and CCN2 results in ERK dependent inhibition of migration

In the search for molecular linkers between haemodynamic dysregulation, the renin-angiotensin system and renal scarring, TGFβ expression was found to be upregulated in glomerular diseases including experimental glomerulonephritis [20] and experimental and human DN within the mesangium [21,22]. Increased glomerular expression of CCN2 has also been reported in a variety of human glomerulopathies, including IgA nephropathy, focal and segmental glomerulosclerosis as well as diabetic nephropathy [23-25]. Increased CCN2 expression has been documented in experimental models of diabetic glomerulosclerosis [26,27]; *in vitro* studies have shown that CCN2 is induced in HMCs by high glucose, AGEs, TGFβ and ROS [6,26,28]. In the streptozotocin (STZ) diabetic mouse model, upregulated expression of CCN2 has been found to be primarily contained to the podocyte in the immediate phases after induction of diabetes [29]; STZ mice that transgenically over-express CCN2 specifically in the podocyte developed more severe proteinuria and mesangial expansion than their control (non-transgenic) littermates [30]. Podocyte derived CCN2 has also recently been proposed to have a paracrine effect on mesangial cells resulting in augmented profibrotic signalling and matrix accumulation independent of active TGFβ [31]. Co-ordinate expression of TGFβ and CCN2 has been previously described [15,32,33]; a potentially complex interplay between autocrine and paracrine factors is likely present in the glomerular microenvironment and contributes to the development of chronic fibrosis. In a recent multi-disease study of the contribution of TGFβ and CCN2 to the development of fibrosis in the kidney, liver and lung, Wang and colleagues demonstrated that CCN2 was both sufficient and necessary to initiate fibrosis in the presence of TGFβ, and vice versa [16].

**Figure 1:**
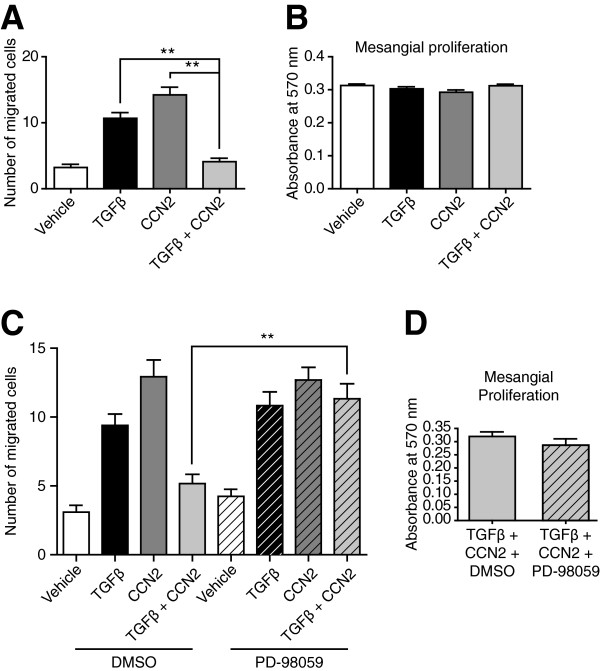
**Co-treatment of HMCs with TGFβ and CCN2 inhibits cell migration in an ERK dependent manner. A**. HMCs were grown to confluence, a wound scratch made and cell migration assessed in response to treatment with TGFβ, CCN2 or both together. Both TGFβ and CCN2 increased cell migration, but together inhibited the migration of cells. **B**. Altered migration was not dependent on cell proliferation, as assessed by MTT uptake. **C**. Inhibition of MEK/ERK signalling with the inhibitor PD-98059 was sufficient to restore migration in cells co-treated with TGFβ and CCN2. **D**. This effect was not dependent on altered cell proliferation. Data representative of n = 3 independent experiments with 10 biological replicates per condition per experiment. **p < 0.01.

In the context of DN, dysregulation of the cytoskeleton is a feature associated with the response of myofibroblast like cells to changes in the composition of extracellular matrices. Modelling of migration in cells provides a useful insight into the effects of TGFβ and CCN2 on the organisation of the cytoskeleton. In human mesangial cells (HMCs) treated with TGFβ or CCN2, the number of cells migrating is increased, in line with previous observations [18,34]. However, co-treatment of HMCs with both growth factors is sufficient to prevent the migration of cells (Figure 1A), with this effect found independent of altered proliferation (Figure 1B). It has previously been reported that decreased activation of canonical Smad signalling in cells treated with both TGFβ and CCN2 is associated with increased non-canonical signalling by ERK MAP kinase [13]. In cells treated with both TGFβ and CCN2, inhibition of MEK/ERK with the selective inhibitor PD-98059 was sufficient to rescue the migration of HMCs (Figure 1C), again independently of altered proliferation (Figure 1D), suggesting a role for ERK in this process.

### Pathway analysis of genes differentially expressed in cells treated with TGFβ and CCN2

**Figure 2:**
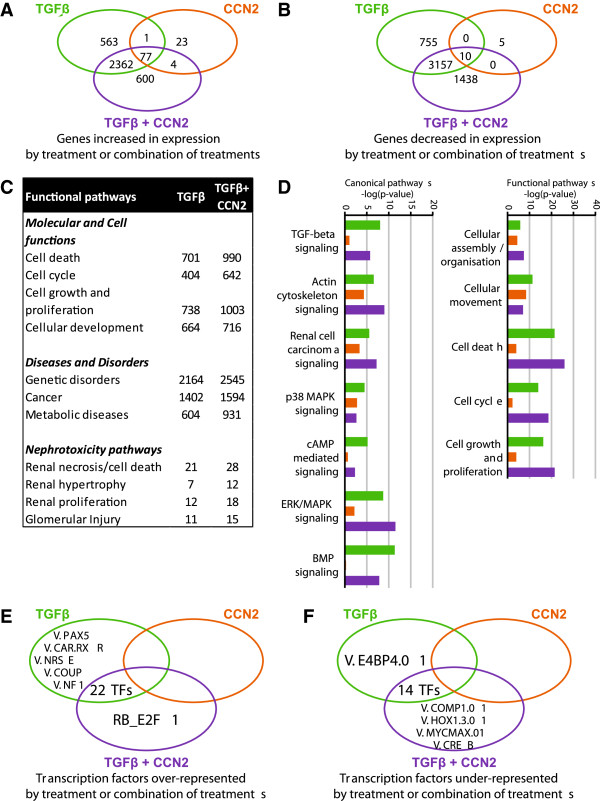
**TGFβ and CCN2 together induce differential gene expression associated with altered functional and canonical pathways and unique transcription factor representation. A**, **B**. Treatment of HMCs with TGFβ, CCN2 or both together induced differential expression of shared and unique subsets of genes. For each condition, n = 3 gene arrays were used with the average determined by GC Robust Multi-array average and the change in gene expression determined using Student’s t-test and Benjamini-Hochberg correction at p < 0.05. **C**, **D**. Pathway analysis with Ingenuity IPA software identified the augmentation of functional pathways associated with the cell cycle, cell growth and proliferation and renal hypertrophy/glomerular injury; canonical pathways including signalling via the TGFβ superfamily and cAMP were decreased in activity while increased ERK/MAPK activity was identified. **E**, **F**. Co-inertia analysis of the promoters of the genes differentially expressed in response to TGFβ, CCN2 or both together identified TFBSs that were over and under-represented in specific gene subsets at p < 0.05.

Altered gene expression in HMCs treated with TGFβ, CCN2 or both together was previously determined by gene array [13]. TGFβ and CCN2 together differentially regulated over 2000 genes in HMCs that were not regulated by either growth factor alone (Figure 2A, 2B). CCN2 on its own only regulated the expression of approximately 120 genes, supporting the hypothesis that this growth factor plays a major role in the integration of signalling by other molecules rather than CCN2 itself regulating gene expression. Using Ingenuity Pathway analysis (IPA) (Ingenuity Systems, http://www.ingenuity.com) we identified that co-treatment of HMCs with TGFβ and CCN2, resulted in augmented expression of genes associated with molecular and cellular functions including cell growth and proliferation, while also enhancing nephrotoxicity associated pathways including proliferation and hypertrophy associated genes (Figure 2C). Analysis of annotated functional pathways (Figure 2D) found a decrease in genes associated with canonical TGFβ/BMP signalling and cAMP signalling with an increase in genes regulated by ERK MAP kinase signalling; supporting a role for divergent activation of canonical and non-canonical signalling in the mesangial cell expansion.

### Identification of an altered transcriptional regulatory signature – E2F1 and CREB are over and under-represented in HMCs co-treated with TGFβ and CCN2

**Figure 3:**
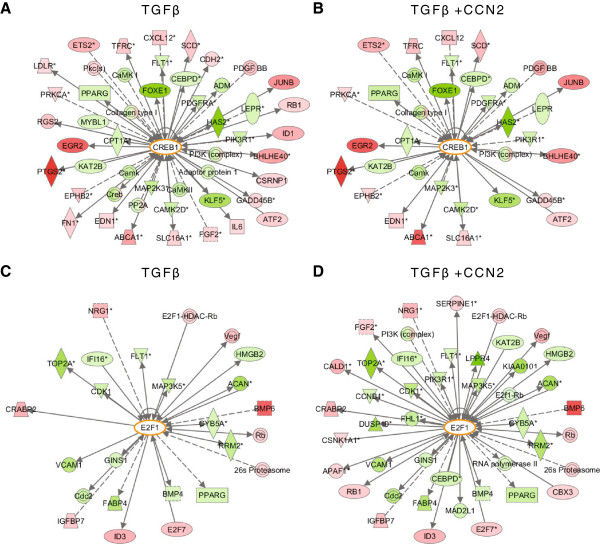
**Altered CREB/E2F1 signalling networks in co-treated HMCs. A**. Pathway analysis with Ingenuity IPA software identified interacting partners of CREB differentially expressed in response to treatment with TGFβ. **B**. In comparison, fewer genes related to CREB signalling were differentially expressed in co-treated cells. **C**. Similarly, pathway analysis identified E2F1 interacting partners in cells treated with TGFβ. **D**. The number of E2F1 partners was enriched by co-treatment with TGFβ and CCN2. Interacting partners coloured pink/red were increased in expression in response to treatment, those coloured green were decreased in expression.

We hypothesised that the differential gene expression profile seen in co-treated HMCs would be dependent on a change in the transcriptional regulatory network of the cells. We interrogated the promoters of the differentially regulated genes to determine common transcription factor binding sites (TFBSs) in the 500 bp region upstream of the transcription start site. Co-inertia analysis of these promoters identified TFBSs that were over or under-represented relative to control HMCs (Figure 2E, 2F, known physiological functions summarised in the table in Additional file 1). Some 22 TFBSs were found increased in occurrence in genes common to HMCs treated with either TGFβ or TGFβ and CCN2 together, while 14 TFBSs were found to be decreased in occurrence. A subset of transcription factors were found to be uniquely regulated by TGFβ and CCN2; two of these transcription factors were selected for further characterisation – E2F1, as the sole transcription factor over-represented by co-treatment and CREB, with an established response to TGFβ and as a key component of cAMP signalling that was identified by pathway analysis to be negatively regulated in cells treated with TGFβ and CCN2. Pathway analysis of both transcription factors confirmed altered expression of associated genes in cells treated with both TGFβ and CCN2 versus TGFβ alone (Figure 3).

### Validation of transcription factor signature – co-treatment of HMCs with TGFβ and CCN2 results in decreased active CREB levels and increased E2F1 activity, which is dependent on ERK signalling

**Figure 4:**
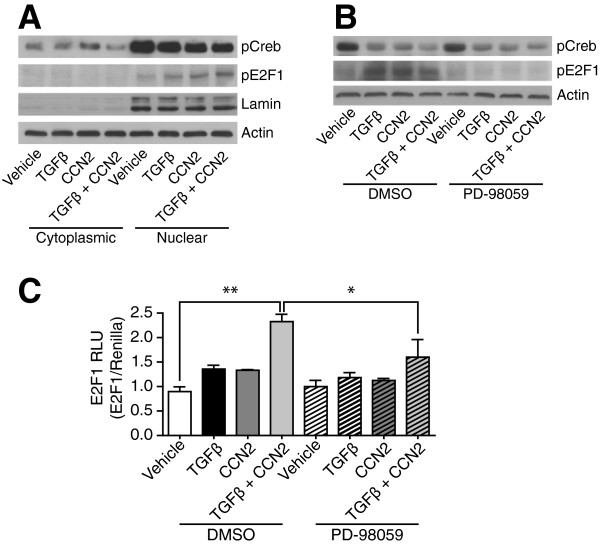
**TGFβ and CCN2 induce differential activation of both CREB and E2F1 in HMCs, inhibition of MEK/ERK decreases E2F1 activation. A**. HMCs were treated with TGFβ, CCN2 or both together, and expression of pCREB and pE2F1 were determined, as well as their localisation within the cell. CREB activation is decreased by co-treatment, while E2F1 activation is increased. The localisation of either transcription factor was unchanged by treatment with either growth factor. **B**. The effect of TGFβ and CCN2 on E2F1 activation was dependent on ERK signalling, inhibition of MEK/ERK prevented activation of the transcription factor. Blots representative of n = 3 independent experiments. **C**. The activation of E2F1 by TGFβ and CCN2 had a transcriptional consequence as assessed by the activation of an E2F1 promoter containing luciferase construct; again this effect was ERK dependent. Data from n = 3 independent experiments with 3 biological replicates per condition per experiment. *p < 0.05, **p < 0.01.

Members of the E2F family play essential roles in the regulation of proliferation by stimulation of S-phase genes [18] and overexpression of E2F1 overcomes the inhibition of cell growth by TGFβ at mid-G1 phase [19]. In diabetes, E2F1 negatively regulates growth of mature pancreatic cells and maintains differentiated pancreatic cell phenotypes [20]; in renal disease, increased E2F1 has been found in proliferating glomeruli in human IgA nephropathy [21]. In the kidney, CREB is reported as a positive and negative regulator of gene expression in disease pathophysiology. In tubular cells it transduces pro-survival signals after oxidant stress [22] and regulates hexosamine induced fibronectin synthesis in mesangial cells [23]. High extracellular glucose and TGFβ stimulate fibronectin production in mesangial cells by cAMP/CREB [24]; use of anti TGFβ antibodies has been reported to decrease CREB phosphorylation and hypertrophy in diabetic glomeruli [25]. Treatment of HMCs, with TGFβ or CCN2, decreased pCREB levels, co-treatment of cells consistently decreased the levels of activated CREB further (Figure 4A). Neither TGFβ nor CCN2 altered the localisation of CREB within the cell, suggesting intermediate transduction of the regulatory signal to already localised nuclear CREB. TGFβ and CCN2 also increased expression of activated E2F1 (Figure 4A) and transcriptional activity of an E2F1 promoter containing luciferase reporter (Figure 4C), demonstrating a transcriptional consequence for genes containing the TFBS. With the preceding observation that inhibition of MEK/ERK signalling restored the ability of HMCs to migrate when treated with TGFβ and CCN2, we investigated whether this was associated with the regulation of E2F1/CREB. It is well established that many growth factors can activate CREB via phosphorylation at its Ser-133 site, with this effect being mediated by protein kinases including MEK/ERK/p38 and Rsk [35]. Attenuation of ERK expression in HMCs with the MEK inhibitor PD-98059, was therefore unsurprisingly found not to alter the phosphorylation state of CREB (Figure 4B). However, inhibition of ERK both restricted the activation of E2F1 (Figure 4B) as well as the activation of E2F1 luciferase (Figure 4C); these data suggest a role for ERK in the regulation of E2F1.

### Identification of an actin-related gene expression profile with stratified occurrence of E2F1 and CREB TFBSs

**Figure 5:**
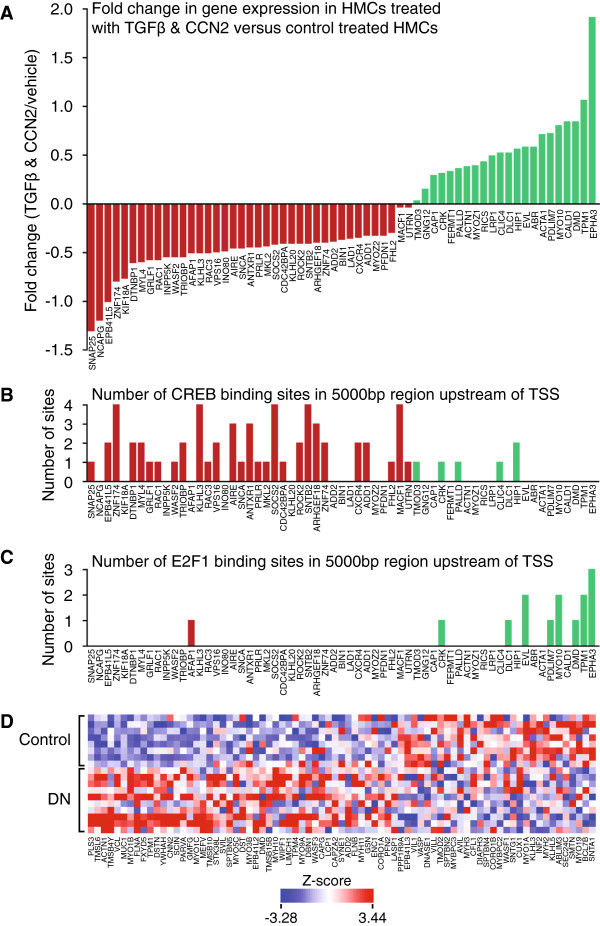
**Actin related genes containing one or more CREB and E2F1 binding sites stratify in expression versus occurrence of transcription factor binding sites; a subset of actin related genes are differentially expressed in DN.** Actin related genes were extracted from each gene set for each condition by gene ontology (GO) annotation search. For each actin related gene, the occurrence of CREB and E2F1 sites were determined in the 5000 bp region upstream of the transcription start site (TSS). **A**. The genes are arranged as fold change in expression for the co-treated dataset versus the control dataset. **B**, **C**. The occurrence of CREB and E2F1 sites were determined, and appear to stratify with change in gene expression, suggesting functional consequence for the presence of these transcription factor binding sites with respect to the change in gene expression induced by TGFβ and CCN2. **D**. A subset of actin related genes from the ERCB biobank illustrate differential gene expression in biopsy samples from patients with DN versus controls (genes differentially expressed at p < 0.05).

CREB and E2F1 TFBSs were under and over-represented (respectively) in HMCs treated with both TGFβ and CCN2; ERK inhibition restored migration and inhibited E2F1. Therefore we examined the role of genes associated with actin cytoskeletal reorganisation and migration containing CREB and E2F1 TFBSs. Gene ontology (GO) annotation was used to address this and extract a subset of genes containing the GO term ‘actin’ which were subsequently examined for CREB and E2F1 sites in the 5000 bp region upstream of the transcription start site. Interestingly, the occurrence of CREB and E2F1 sites varied by gene expression profile: genes that decreased in expression in co-treated cells relative to control cells contained more CREB binding sites, while positively regulated genes contained more E2F1 binding sites (Figure 5A-5C). This data suggests a role for both transcription factors in the altered migratory responses seen upon co-treatment. The table in Additional file 2 summarises the physiological functions ascribed to the actin cytoskeletal genes found to be differentially regulated. These genes include a number of master regulators associated with cell structural dynamics including adhesion, migration, cytoskeletal reorganisation and actin dynamics. In DN, the expression of both TGFβ [22] and CCN2 [24] are increased; altered actin and cytoskeletal dynamics is a feature of the disease [36,37]. Data from the European Renal cDNA bank (ERCB) illustrates that genes associated with actin reorganisation are altered in DN (Figure 5D); it seems likely that the altered actin cytoskeletal dynamic profile observed *in vitro* is re-capitulated in disease.

### Differential regulation of hypertrophic genes in HMCs treated with TGFβ and CCN2

The earliest gross pathological renal feature of diabetes mellitus is generalised renal growth [26]. Tubuloepithelial hypertrophy and tubular basement membrane thickening occur early while glomerular growth processes have been found to always precede glomerulosclerosis in experimental models of DN [27]. The association between growth and fibrogenesis may be due to the fact that similar networks of growth factors and cytokines that induce cellular hypertrophy also stimulate extracellular matrix synthesis and deposition [6]. TGFβ is one of the principal effectors of glomerular hypertrophy, with increased levels of the growth factor found early in multiple models of DN, in parallel with increased kidney weight [28]. In line with its role as a mediator of TGFβ, CCN2 is also reported to transduce hypertrophic TGFβ signalling [29]. With TGFβ and CCN2 being reported as both pro-migratory and pro-hypertrophic, we hypothesised that the altered phenotype may represent an enhanced hypertrophic response. Using Ingenuity pathway analysis, we extracted a panel of hypertrophy associated genes differentially regulated by TGFβ and CCN2, but inconsistent expression profiles provided inconclusive evidence supporting a role for hypertrophy in the change in migratory response (summarised in the table found in Additional file 3).

### Co-treatment driven Cdc42 activation and signalling in HMCs and in disease

Cdc42, a member of the Rho family of GTPases, has been shown to play a role in cell motility and migration [38]. Cdc42 is active towards the front end of migrating cells; inhibition or global activation of Cdc42 results in disrupted directionality of migration [39]. To determine if an alteration of Cdc42 occurred in cells treated with TGFβ and CCN2, the activity of the protein was determined using an active Cdc42 pull-down assay (Additional file 4: Figure S1A). In cells treated with TGFβ and CCN2, Cdc42 activation was increased (Additional file 4: Figure S1A); in addition the phosphorylation of the Cdc42 effector kinases Pak1/2 was also increased (Additional file 4: Figure S1B). Pathway analysis of co-treatment regulated genes identified differential expression of interacting partners of Cdc42 and Pak1 (Additional file 4: Figure S1C), suggesting the altered migratory capacity in co-treated cells may be associated with differential Cdc42/Pak signalling. Analysis of ERCB data identified a cohort of Cdc42 interacting proteins that were altered in expression between control and DN patients (Additional file 4: Figure S1D), supporting a disease role for this signalling context.

## Conclusions

The data shown here examines the transcriptional basis of how TGFβ and CCN2 together alter the migratory capacity of HMCs, determining that there is a role for CREB and E2F1 in regulating the expression of actin related genes that contribute towards the ability of cells to move. From their known functions in the regulation of cell cycle transition [40], hypertrophy [41] and proliferation [42], it is likely that these functions are also part of the mechanism by which CREB and E2F1 play roles in cell migration; in HMCs these processes were identified from gene expression data to be modified by the presence of both TGFβ and CCN2. A further role for Cdc42 as a major effector of polarisation and cytoskeletal organisation was also found, with expression of the active form of this protein being regulated by TGFβ and CCN2 together. In the context of DN, a better understanding of the interplay between CCN2, a known effector of cytoskeletal reorganisation and TGFβ will undoubtedly enhance our understanding of the dysregulation of actin cytoskeletal processes in this kidney disease.

## Methods

### Cell culture

Primary HMCs from Clonetics (Lonza) were cultured in MCDB-131 (GIBCO, Invitrogen), HEK-293 T/17 embryonic kidney cells (ATCC) were cultured in DMEM (Lonza); both media were supplemented with 10% fetal bovine serum (FBS), 100 units/ml penicillin, 1 mg/ml streptomyocin and 2 mM L-glutamine (all from Invitrogen). Confluent cells were growth arrested for 24 hours prior to treatment in media with penicillin/streptomyocin and L-glutamine alone. Cells were cultured with TGFβ (10 ng/ml, PromoKine), CCN2 (25 ng/ml, Fibrogen Inc.) or both together for 24 hours. The inhibitor of MEK, PD-98059 (Calbiochem), was added at a final concentration of 10 μm 1 hour prior to stimulation.

### Scratch wound migration assay

Cells were cultured on 12 well plates to confluency and growth arrested. Scratches were made to the cell monolayer using a pipette tip. The media was then replenished with media containing growth factors as indicated. After 24 hours, the cells were fixed with 3.7% paraformaldehyde (Electron Microscopy Sciences) and stained with Hoechst 33342 (Invitrogen). The number of nuclei migrating into the wound was assessed on a Zeiss Axiovert microscope for at least 10 different wounds per condition.

### MTT proliferation assay

Cells were counted, plated onto 96 well plates and proliferated for 24 hours before being growth arrested and treated for 24 hours. 10 μl of MTT was added (final concentration 500 μg/ml) and the plate was incubated at 37°C for 3 hours. Formation of formazan crystals was confirmed and 100 μl of DMSO was added to solubilise. Absorbance was determined at 570 nm.

### E2F1 luciferase assay

The luciferase construct pGL2-AN-E2F1 (Addgene, #20950), containing the promoter of the E2F1 gene and a Renilla luciferase construct were transfected into HEK-293 cells for 24 hours. The cells were growth arrested and treated for 24 hours. Luciferase activity was assessed with a dual-luciferase reporter kit (Promega).

### RNA isolation and human genome array

Total RNA from HMCs was reverse transcribed, fragmented and hybridised to an Affymetrix human genome U133 plus 2.0 array (Affymetrix). Data from replicates of three arrays per condition were normalised by GC Robust Multi-array average before a linear model was applied and differentially expressed genes were identified using a modified Student’s t-test and Benjamini-Hochberg correction at p < 0.05. A complete list of all genes and their expression levels for each of TGFβ, CCN2 and both together can be found in Additional files 5, 6 and 7 (respectively).

### Preparation of cellular protein extracts including nuclear fractionation and Western blotting

Protein extracts were prepared in lysis buffer containing Tris/HCl, sodium deoxycholate, NaCl, EGTA, NaF, Igepal CA-630, PMSF and protease/phosphatase inhibitor cocktails (all from Sigma). For fractionated samples, cells were lysed with an NE-PER nuclear and cytoplasmic extracts kit (Pierce) according to the manufacturer’s instructions. Protein was quantified by Bradford assay (Bio-Rad Laboratories). Samples were resolved by SDS-PAGE, transferred to membrane, blocked in phosphate buffered saline (PBS) containing 0.1% Tween 20 and 5% (w/v) skimmed milk and incubated overnight with anti-phospho-CREB (Cell Signaling), anti-phospho-E2F1 (Abcam) or Phospho-PAK1/2 (Cell Signaling) at 4°C. Secondary HRP-conjugated antibodies (Cell Signaling) were incubated at room temperature for 1 hour.

### Activated Cdc42 pulldown assay

Cdc42 activation was determined using an Active Cdc42 Pull-Down and Detection Kit (Thermoscientific) as per manufacturer’s instructions. Briefly cells were washed, lysed, centrifuged at 4°C for 15 minutes, followed by determination of protein concentration. EDTA and either GTPγS or GDP were added and incubated for 15 minutes at 30°C. The reaction was stopped by the addition of MgCl2. The lysate was then added to a spin column containing a Glutathione Swell Gel Disc and GST-PAK-1-PBD, incubated for 60 minutes at 4°C, centrifuged at 7200 g for 30 seconds and washed three times with PBS. SDS containing β-mercaptoethanol was added to the sample, collected by centrifugation and resolved by SDS-PAGE. Expression of the pulldown protein and total protein was determined using an anti-Cdc42 antibody (Cell Signaling).

### Promoter analysis for differentially represented transcription factor binding sites

Unsupervised co-inertia analysis (CIA) was used to extract differentially represented transcription factor binding sites in the promoters of genes regulated by TGFβ, CCN2 or both together in HMCs. A combined total of 1236 binding sites were used with a position specific matrix of 0.85 applied to each gene set to produce a motif/gene matrix for CIA [43].

### Statistical analysis

Graphs are expressed as mean +/− standard error of the mean (s.e.m.) Analysis was by one way ANOVA with post hoc Tukey’s test or Student’s t-test as appropriate for the number of groups; analysis was carried out with GraphPad Prism (GraphPad).

## Competing interests

The authors declare that they have no competing interests.

## Authors’ contributions

NF and HOD contributed to the design of the study and carried out the experiments. DK analysed the promoters of the actin related genes, SM carried out the unsupervised CIA promoter analysis. APM, FM, GM and CG participated in the study design and coordination. JC conceived the study and coordinated the research. NF and JC drafted the manuscript. All authors read and approved the final manuscript.

## Supplementary Material

Additional file 1 **Summary of transcription factors with differentially represented Transcription Factor Binding Sites in gene subsets.** Known physiological functions of transcription factors with TFBSs found to be differentially represented at p < 0.05 by co-inertia analysis. Click here for file

Additional file 2 **Summary of actin related genes with CREB or E2F1 binding sites.** Genes containing GO annotation of ‘actin’ and containing one or more binding sites for CREB or E2F1. Click here for file

Additional file 3 **Pathway analysis identified hypertrophic genes.** Genes identified by IPA pathway analysis with functional role in cell hypertrophy. Click here for file

Additional file 4 **Activation of Cdc42 signalling in co-treated cells and expression of associated interacting partners.** Figure S1 **A**. A Cdc42 pulldown assay was used to determine expression of activated Cdc42 in HMCs in response to TGFβ, CCN2 and both together. Cdc42 activation was only responsive to treatment with both growth factors. **B**. Downstream signal transduction by the Cdc42 effector proteins PAK1 and PAK2 was also increased by co-treatment of cells. **C**. Pathway analysis of differentially activated genes identified that a number of Cdc42 interacting proteins (left) and PAK1 interacting proteins (right) were differentially expressed by co-treatment of HMCs with TGFβ and CCN2. Interacting partners coloured pink/red were increased in expression in response to treatment, those coloured green were decreased in expression. Figure S1 **D**. Analysis of the expression of these genes from the ERCB biobank demonstrates that Cdc42 interacting partners are differentially expressed in DN, suggesting disease relevance of the HMC phenotype. Click here for file

Additional file 5 **Genes regulated in HMCs by TGFβ.** Array genes differentially regulated by TGFβ at p < 0.05. Click here for file

Additional file 6 **Genes regulated in HMCs by CCN2.** Array genes differentially regulated by CCN2 at p < 0.05. Click here for file

Additional file 7 **Genes regulated in HMCs by TGFβ and CCN2.** Array genes differentially regulated by TGFβ and CCN2 at p < 0.05. Click here for file
