# Supplementary figures and images for: TGFβ and CCN2/CTGF mediate actin related gene expression by differential E2F1/CREB activation
Source: BMC Genomics. 2013 Aug 1;14:525. doi: 10.1186/1471-2164-14-525 (PMC3765338; doi:10.1186/1471-2164-14-525)

### Figure 6

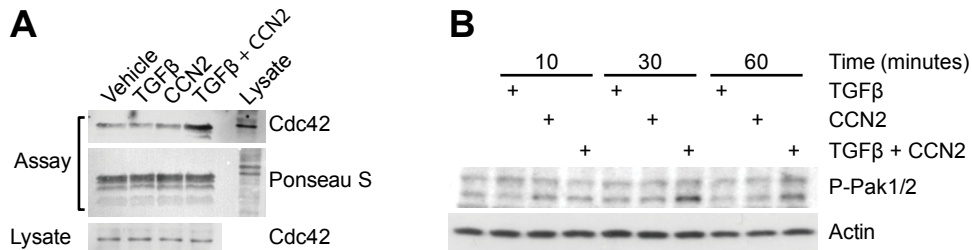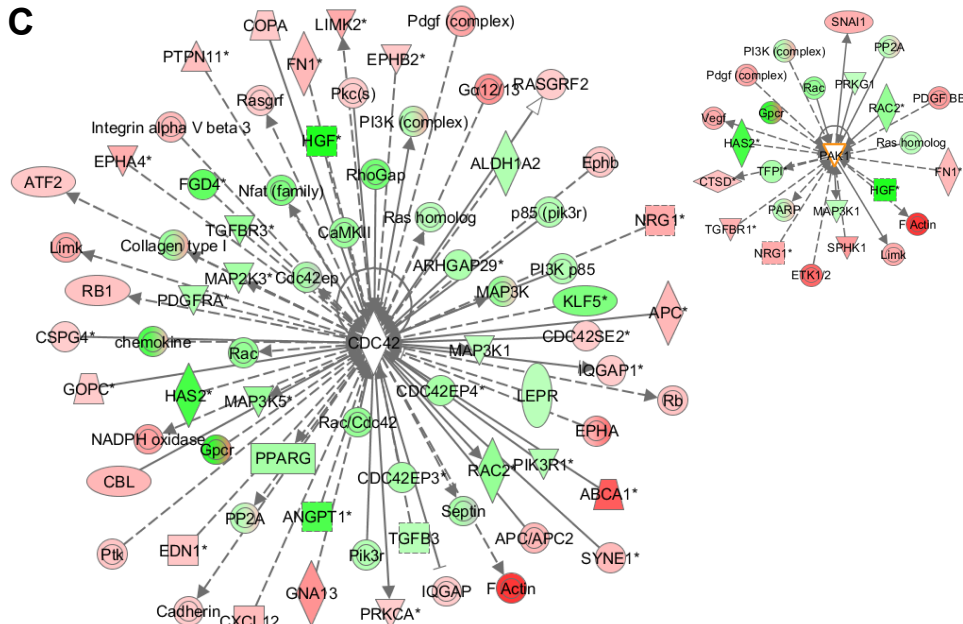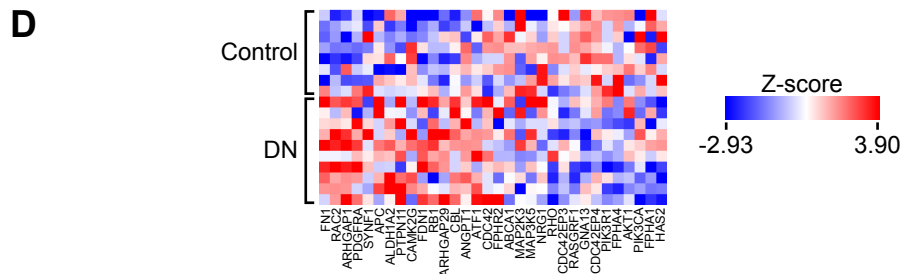

Supplement: Additional file 7 — Genes regulated in HMCs by TGFβ and CCN2. Array genes differentially regulated by TGFβ and CCN2 at p < 0.05. [file 1471-2164-14-525-S7.pdf]
